# Exploring the Relationship Between Problematic Internet Use (PIU) and Fear of Missing Out (FoMO) Among Adolescents

**DOI:** 10.3390/ijerph23050605

**Published:** 2026-05-03

**Authors:** Ethan H. Yu, Chelsea Olson, Megan A. Moreno

**Affiliations:** Department of Pediatrics, School of Medicine and Public Health, University of Wisconsin–Madison, Madison, WI 53792, USA; ehyu@wisc.edu (E.H.Y.); cjolson5@wisc.edu (C.O.)

**Keywords:** problematic internet use, fear of missing out, gender differences, adolescents

## Abstract

**Highlights:**

**Public health relevance—How does this work relate to a public health issue?**

- Adolescent Problematic Internet Use (PIU) and Fear of Missing Out (FoMO) are increasingly prevalent and have been linked to adverse mental, social, and academic outcomes.
- This study examines how PIU and FoMO interact during adolescence, a critical development period marked by heightened vulnerability to digital overuse.

**Public health significance—Why is this work of significance to public health?**

- Findings highlight that the relationship between PIU and FoMO is not uniform, but varies by gender.
- By identifying different patterns of risk, this work advances understanding of how maladaptive digital behaviors manifest across subgroups of adolescents.

**Public health implications—What are the key implications or messages for practitioners, policy makers and/or researchers in public health?**

- Prevention and intervention efforts may benefit from being tailored to both gender and level of PIU severity, rather than adopting a one-size-fits-all approach.

**Abstract:**

(1) Background: Previous studies have shown that increased Problematic Internet Use (PIU) is associated with higher Fear of Missing Out (FoMO). However, the role of gender in this association remains unclear. Evidence suggests that males and females may experience PIU and FoMO differently, warranting further examination of gender differences. (2) Methods: In this cross-sectional study, a total of 4370 U.S. adolescents aged 12–17 years (47% female) were recruited through Qualtrics research panels and completed a national online survey on adolescent health and technology. Demographic information collected included age, race, and gender. PIU was measured using the validated three-item Problematic and Risky Internet Use Screening Scale (PRIUSS-3), while FoMO was measured using a validated 10-item FoMO scale. Multiple linear regression analysis examined the relationship between PIU and FoMO, including an interaction term between PIU and gender to assess the potential gender moderation effect while adjusting for age and race. (3) Results: Gender significantly moderated the relationship between PIU and FoMO, indicating that males experience a stronger association between PIU and FoMO compared to females. While higher PIU scores were significantly associated with higher FoMO for both males and females, each unit increase in PIU corresponded to a 0.15 point increase in FoMO for females (*SE* = 0.01, *p* < 0.01), and an additional 0.03 point increase for males (*SE* = 0.01, *p* < 0.01). (4) Conclusion: These findings illustrate the complex relationship between PIU and FoMO, highlighting the importance of gender-specific strategies and targeted interventions for promoting healthy Internet use.

## 1. Introduction

Over 97% of teens have reported being on the Internet every day, and the proportion of adolescents reporting being online “almost constantly” has doubled from 24% in 2014–2015 to 46% in 2022 [1]. The rapid growth of social media and internet-based communication has transformed how adolescents connect, share, and engage with others [2]. Recent research indicates that adolescents are a particularly vulnerable group to maladaptive internet use, spending more time online than other age groups and potentially experiencing adverse consequences in their personal, social, and academic outcomes [3]. Correspondingly, there has been an increase in problematic forms of online engagement among adolescents including excessive use of social media, online gaming, and other internet-based content. These behaviors are commonly understood as manifestations of Problematic Internet Use (PIU) which is defined as internet use that is excessive, risky, or impulsive and results in adverse physical, emotional, social, or functional consequences [4]. PIU has been consistently associated with adverse mental health outcomes such as depression and anxiety [2,4,5,6]. Notably, these same mental health outcomes have also been linked to higher levels of FoMO, suggesting overlapping psychological vulnerabilities across maladaptive internet use and FoMO [7].

Adolescence represents a developmental period of heightened vulnerability to PIU, driven by ongoing emotional, cognitive, and social development [6]. Adolescents who experience difficulties with emotion regulation may be particularly susceptible to PIU. Consistent with this, PIU has been conceptualized as a behavioral addiction characterized by loss of control, preoccupation, and continued use despite negative outcomes [8]. During adolescence, heightened sensitivity to peer influence and an increasing reliance on online communication for social validation may further amplify vulnerability to problematic online engagement [9]. Reflecting these developmental risk factors, prevalence estimates suggest a substantial proportion of adolescents, up to 44%, may meet criteria for PIU [10]. Importantly, PIU has been linked to a range of negative mental health outcomes, including mood and anxiety disorders, poorer sleep quality, decreased academic performance, and depression [9], many of which overlap conceptually with emerging research on the Fear of Missing Out (FoMO).

As digital communication becomes key to adolescents’ social lives, the Fear of Missing Out (FoMO) has emerged as a prominent construct in efforts to understand adolescents’ online experiences [11]. FoMO is particularly salient during adolescence, a developmental period marked by heightened sensitivity to peer relationships and social belonging [11]. Przybylski et al. (2013) describe FoMO as a persistent concern about being excluded from meaningful experiences others are having, which motivates individuals to frequently monitor and stay engaged with others’ social activities [11]. According to Self-Determination Theory, FoMO is conceptualized as a disruption in self-regulation that emerges when individuals experience unmet psychological needs. Specifically, deficiencies in relatedness, autonomy, and competence may contribute to dysregulated behaviors and increased sensitivity to social exclusion [11]. Within increasingly digital social environments, this vulnerability may be intensified by heightened social comparison and constant awareness of others’ activities, reinforcing anxieties and unmet needs about exclusion or falling behind that characterize FoMO.

Consistent with this framework, as adolescents experience more persistent and interactive online involvement, they are increasingly exposed to social information that highlights potential exclusion, even when such exclusion is minor or perceived rather than actual [12,13]. This intensified exposure to socially comparative content may heighten FoMO by amplifying concerns about being left out or falling behind peers [12,13]. Platform features such as notifications, likes, and messages function as intermittent rewards, reinforcing repeated checking behaviors, while missed notifications may elicit discomfort or anxiety [2,14]. Over time, these reinforcement patterns may contribute to compulsive use, loss of control, and difficulty disengaging from online platforms, which are core features of PIU [9,15]. In line with the Compensatory Internet Use Model, adolescents may engage in excessive internet use to cope with FoMO-related distress. However, increased online engagement may simultaneously heighten exposure to social comparison and cues of exclusion, further intensifying FoMO. This suggests a reciprocal, self-reinforcing cycle in which PIU and FoMO mutually exacerbate one another over time [16,17].

Despite growing recognition of the relationship between PIU and FoMO, the role of gender in shaping these dynamics remains unclear. Prior research has produced mixed findings relating to PIU, with many studies reporting higher levels among males, others finding higher levels among females, and still others observing no significant gender differences [18,19,20,21]. Patterns often vary by type of online activity, such as gaming, social media use, or online relationships [18,19]. Similarly, evidence regarding gender differences in FoMO is inconsistent, reflecting findings of higher FoMO among males, no significant gender differences, and, in some cases, higher FoMO among females [22,23,24]. Even fewer studies have examined whether gender moderates the association between PIU and FoMO, and existing evidence remains mixed or inconclusive [25,26].

Accordingly, the present study examines whether gender moderates the association between PIU and FoMO among adolescents using a large, nationally representative sample of U.S. adolescents.

The present study aims to clarify the moderating role of gender in the association between PIU and FoMO among adolescents. Specifically, we examine:

1. Whether PIU and FoMO are positively associated, consistent with a bidirectional, self-reinforcing model of maladaptive online engagement.
2. Whether gender moderates this relationship, such that the strength or direction of the association differs for males and females.

Although the data was collected in 2019, the findings remain relevant because adolescents continue to spend increasing amounts of time online, and pre-pandemic patterns provide an important baseline for understanding how PIU and FoMO may have changed during and after the COVID-19 pandemic.

## 2. Materials and Methods

### 2.1. Study Design

A national online Qualtrics survey (Qualtrics LLC, Provo, UT, USA; www.qualtrics.com) was administered cross-sectionally during February and March, 2019. This study represents a secondary analysis of previously collected data. The Institutional Review Board (IRB) at the University of Wisconsin approved the data used in this study and participation was voluntary. The target population and inclusion criteria were adolescents aged 12–17 years who were United States residents and English-speaking, while exclusion criteria included individuals outside of this age range, non–U.S. residents, and those who did not speak English. Parameters were established for Qualtrics to recruit a quota-based sample from their existing panels consistent with race/ethnicity and gender distributions representative of the U.S. census population for this age group. Previous studies have shown that online survey approaches using tools such as Qualtrics can use quota-based sampling to achieve demographic attributes that are typically within a 10% range of their corresponding values in the U.S. population [27]. There is a strong and growing literature around the use of Qualtrics to recruit samples in the U.S., including studies of adolescent technology use [28,29]. A Qualtrics survey manager recruited adult participants who indicated they had adolescent children who met eligibility criteria during the study period; eligible parents completed informed consent, after which adolescents provided assent prior to participation, with both required for inclusion. Participants received compensation through Qualtrics panel points-based rewards. Responses identified as invalid during data cleaning were excluded from the final analytic sample.

### 2.2. Measures

PIU was measured using the validated Problematic and Risky Internet Use Screening Scale Brief (PRIUSS-3), a shortened clinical screening version of The Problematic and Risky Internet Use Screening Scale (PRIUSS-18) [30]. The abbreviated scale includes three items selected from the original PRIUSS-18 to capture social impairment, emotional impairment, and risky/impulsive internet use. The statements included “How often do you experience increased social anxiety due to your internet use?”, “How often do you feel withdrawal when away from the internet?” and “How often do you lose motivation to do other things that need to get done because of the internet”. Participants rated each statement on a 5-point Likert scale ranging from 0 (“Never”) to 4 (“Very often”). The total PIU score ranged from 0–12, with higher scores indicating greater PIU, and was calculated by summing across all three items. The Cronbach’s alpha for these three items was 0.872.

Fear of Missing Out (FoMO) was measured through the widely used and validated 10-question instrument, which assessed a teen’s fear of missing out on activities their peers engage in [11]. The scale consists of 10 items assessing the extent to which individuals fear missing rewarding social experiences or opportunities that others might be having, such as “I fear others have more rewarding experiences than me” and “I get worried when I find out my friends are having fun without me”. Responses were recorded on a 5-point Likert scale from 1 (“Not at all true of me”) to 5 (“Extremely true of me”). The total FoMO score ranged from 1–5, with higher scores indicating greater FoMO, and was calculated by averaging across all items. The Cronbach’s alpha for these 10 items was 0.926.

Adolescent participants provided demographic information including age, gender, and race.

### 2.3. Data Analysis

All analyses were conducted using R version 4.3.0. Only adolescents who identified as male or female were included in the analysis. Age was classified as a categorical variable using years of age. Race was categorized as three groups: White/Caucasian, Black/African American, and other. Groups were selected due to being the most prevalent in the sample. Descriptive statistics were calculated for all study variables, including counts and frequencies for categorical variables and means with standard deviations for continuous variables. FoMO and PIU scores often show non-normal skewed distributions in social media studies. However, with large sample sizes, these violations of normality become less critical for parametric tests. Gender differences were then tested for PIU and FoMO using *t*-tests. A univariate linear regression model was first used to examine the unadjusted association between PIU and FoMO. Finally, a moderation analysis was conducted using a multiple linear regression model to examine the adjusted association between PIU and FoMO, as well as test whether this association differed by gender. This was assessed by including PIU, gender, and the interaction term between PIU and gender, with the model also adjusted for age and race. Statistical significance was defined as *p* < 0.05. Scatter plots, residual plots, and partial residual plots were used for model diagnostics.

## 3. Results

A total of 4370 adolescents aged 12–17 years were included in the analysis. The mean age of participants was 14.62 years (*SD* = 1.68). Among these participants, 2314 (53.0%) identified as male, and 2056 (47.0%) identified as female. Regarding race, 2953 (67.6%) were identified as white, 658 (15.1%) as black, and 759 (17.4%) as other (Table 1).

The mean PIU score was 4.74 (*SD* = 3.50), suggesting large variability in PIU across participants. Males reported a mean PIU score of 4.87 (*SD* = 3.65), and females reported a mean PIU score of 4.59 (*SD* = 3.31); significantly higher PIU was observed in males than females (*t*(4366) = 2.70, *p* = 0.0069, *d* = 0.08) in this sample. The mean FoMO score was 2.39 (*SD* = 0.99), indicating moderate levels of FoMO. Males reported a mean FoMO score of 2.40 (*SD* = 1.04) and females reported a mean FoMO score of 2.38 (*SD* = 0.93); no significant gender differences were found for FoMO (*t*(4368) = 0.40, *p* = 0.6875, *d* = 0.02) (Table 2).

The unadjusted model revealed higher PIU was significantly associated with higher FoMO, with each unit increase in PIU corresponding to a 0.17 increase in FoMO (*SE* = 0.003, *p* < 0.001). This finding was also confirmed in the multivariable model. Further in the multivariable model, age was not significantly associated with FoMO, thus there were relatively consistent FoMO levels across the age ranges. Racial differences were observed, with white participants having FoMO scores 0.12 points higher than participants identifying as other races (*SE* = 0.03, *p* = 0.0003), while Black participants had scores 0.11 points higher than those of other races (*SE* = 0.04, *p* = 0.0074) (Table 3). The final multivariable model explained a moderate proportion of variance in FoMO (adjusted *R*^2^ = 0.37), indicating that additional factors beyond the included covariates also may contribute to FoMO levels.

**Figure 1:**
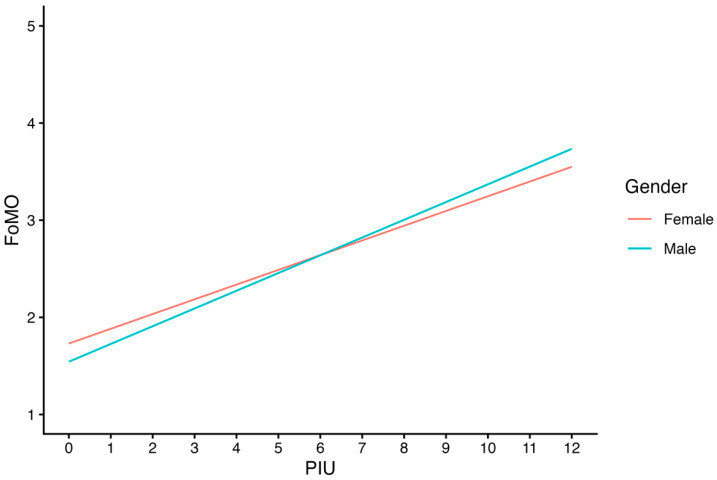
Regression Model Fitted Line for Gender Groups.

The interaction between PIU and gender was statistically significant, indicating that gender moderated the association between PIU and FoMO. Male adolescents, on average, were estimated to have lower FoMO scores than females when their PIU scores were 6 or lower, but higher FoMO scores than females when their PIU scores were 7 or higher (Figure 1). Although the positive association was observed for both genders, the effect was slightly larger among male than female adolescents. Among females (reference group), the effect of PIU on FoMO was 0.15, which means each unit increase in PIU was associated with a 0.15 point increase in FoMO (*SE* = 0.01, *p* < 0.001), while among male adolescents, each unit increase in PIU corresponded to an additional 0.03 point increase in FoMO from the PIU * gender interaction (*SE* = 0.01, *p* < 0.001) (Table 3, Figure 1). In other words, each unit increase in PIU was associated with a 0.18 point increase in FoMO for males. The additional 0.03 for males represents a 20% increase in slope from females.

## 4. Discussion

### 4.1. Summary of Findings

A positive association between the Problematic Internet Use (PIU) and Fear of Missing Out (FoMO) emerged in this U.S. nationally representative sample, indicating that adolescents with higher PIU reported stronger FoMO. Consistent with theoretical frameworks linking maladaptive online engagement to upwards social comparison, this finding aligns with prior work suggesting that concerns about social exclusion or mixed experiences may be closely intertwined with PIU [12]. These findings are also consistent with psychological models that conceptualize PIU and FoMO as part of a self-reinforcing cycle. People high in FoMO are more likely to repeatedly check social platforms, messages, or updates to relieve anxiety about being excluded or uninformed. Heavy or dysregulated internet use, especially social media, exposes individuals to constant social comparison, highlight reels, and real-time updates. This can amplify perceptions that others are having rewarding experiences without them, thereby increasing FoMO [16,17].

Gender differences further contextualized the observed association between PIU and FoMO. Unadjusted comparisons indicated that males reported significantly higher mean PIU scores than females. Previous research has illustrated inconsistent conclusions, our findings are similar to studies concluding males experience higher PIU than females [18,20]. In contrast, no gender differences in FoMO were found in our sample using the unadjusted comparisons, aligning with several prior studies concluding null gender differences despite mixed results in broader literature [24]. However, after adjusting for PIU and demographic covariates in a regression model, the gender difference in FoMO scores was found to vary as a function of PIU levels. The findings in our sample suggest that males may experience elevated levels of FoMO only when PIU scores were high; when PIU level was low, females exhibited higher levels of FoMO than males. The inconsistent or null results found by prior research on adolescent gender differences in FoMO may be explained by the connection to PIU levels, the moderation effect of gender was not considered in previous literature.

In addition, the association between PIU and FoMO was noticeably stronger in the male group. For each unit increase in PIU, male FoMO increased by 0.18 while female FoMO increase by 0.15. The 20% (0.03/0.15) increase indicated that FoMO was more sensitive to change in PIU for male adolescents. The large effect size of PIU*gender interaction indicates that the relationship between PIU and FoMO is strongly moderated by gender. This finding was in line with Swinkels et al., 2025, a longitudinal study on Dutch college students that found a stronger between-persons association for PIU and FoMO in males than in females, while disagreeing with Rozgonjuk, Blinka et al., 2023, which found stronger correlations between PIU and FoMO for females [25,26]. Such discrepancies across studies may be caused by differences in developmental stage, cultural context, or dominant forms of internet engagement. College students and adolescents could have very different emotion and behavioral patterns. One possible explanation is that male adolescents may be less likely than females to engage in offline emotional self-disclosure, which could increase reliance on online environments when experiencing social concerns [31]. Additionally, gendered patterns of internet use such as greater engagement in gaming among males and more socially oriented use among females may uniquely shape how PIU relates to perceptions of social exclusion [32]. Future research should explore these possibilities. Although exploratory, these findings highlight how males and females experience and engage with digital platforms in unique ways, which could influence how problematic use is associated with emotional and motivational outcomes such as FoMO.

### 4.2. Limitations

There are several limitations to the present study. First, the cross-sectional design prevents conclusions about causal directionality or bidirectional relationships between PIU and FoMO. Prior research suggests that FoMO may lead to increased PIU, that PIU may heighten FoMO, and that these processes may reinforce one another in a cyclical manner [16,17]. Second, all variables were self-reported, which may introduce bias or inaccuracies due to adolescents’ perceptions of their own behavior. Third, the sample was drawn from a Qualtrics panel and recruited through parents, which may introduce sampling bias and limit the representativeness of the findings, as participants in online panels may differ systematically from the broader adolescent population. Fourth, the model was limited to two gender categories and three racial groups, and future work could expand it to include a broader range of identities. Fifth, the study did not account for specific types of internet use (e.g., social media platforms, gaming, or messaging), which may differentially relate to PIU and FoMO and provide more nuanced insight into these associations. Finally, the data for this study were collected prior to the COVID-19 pandemic and may be understood as capturing adolescents’ experiences with internet use and FoMO in a pre-pandemic context. The COVID-19 pandemic substantially reshaped adolescents’ daily routines, social interactions, and reliance on digital communication, likely altering both levels of PIU and FoMO. As such, the findings may not fully reflect adolescents’ present digital experiences, but rather provide an important baseline against which post-pandemic patterns can be compared. Given the substantial increase in adolescents’ screen time and digital social engagement during and after the pandemic, it is possible that the strength of the association between PIU and FoMO, and the role of gender as a moderator, may be more pronounced in current populations.

### 4.3. Future Directions

Future research should consider longitudinal designs to examine how PIU and FOMO influence one another over time, including whether these processes operate bidirectionally or reinforce one another across developmental periods. Building on the present study’s pre-pandemic data, future work could also examine whether and how the relationship between PIU and FoMO has changed in the post-pandemic period. Additionally, experimental or ecological momentary assessment (EMA) techniques could help capture adolescents’ digital experiences in real time. Studies should further explore gender differences, including whether different types of online activity such as gaming, social networking, or video-based platforms shape PIU and FoMO dynamics differently for males and females. Finally, investigating interventions such as parental guidance, digital literacy, and emotion regulation skills may help identify mechanisms that reduce the risk of adolescents developing problematic patterns of internet use.

## 5. Conclusions

In conclusion, this study identified a clear positive association between Problematic Internet Use (PIU) and Fear of Missing Out (FoMO) among U.S. adolescents, such that higher levels of PIU were associated with greater FoMO. This finding supports prior theoretical and empirical work suggesting that maladaptive internet use is closely tied to concerns about social exclusion and missed experiences. Gender also emerged as an important moderating factor, with males reporting higher overall PIU and a stronger association between PIU and FoMO than females, while gender differences in FoMO were not fixed, but were rather contingent on PIU levels. These findings align with prior research indicating that male and female adolescents experience PIU and FoMO in different ways due to differences in emotional self-disclosure and internet use patterns, which may shape how problematic use translates into emotional and motivational outcomes.

Taken together, these results highlight the importance of examining PIU and FoMO simultaneously rather than in isolation. Interventions aimed at reducing problematic internet behaviors in adolescents may benefit from addressing underlying fears related to social connection and exclusion, while also considering gender-specific patterns of online engagement. By clarifying how PIU and FoMO interact during adolescence, this study contributes to a growing body of literature seeking to better understand the psychosocial mechanisms underlying adolescent digital well-being and informs future efforts aimed at promoting healthier relationships with internet use.

## Figures and Tables

**Table 1:** Demographic characteristics.

Characteristics	n	%
Gender		
Female	2056	47.0
Male	2314	53.0
Race		
White/Caucasian	2953	67.6
Black/African American	658	15.1
Other	759	17.4
Age (years)		
12	582	13.3
13	752	17.2
14	770	17.6
15	710	16.3
16	764	17.5
17	792	18.1
Age (years)	mean	*SD*
	14.62	1.68

**Table 2:** Gender Comparison using *t*-test.

Measurement	All (n = 4370)	Male (n = 2314)	Female (n = 2056)	*p*-Value
	Mean (SD)	Mean (SD)	Mean (SD)	
PIU	4.74 (3.50)	4.87 (3.65)	4.59 (3.31)	0.0069
FoMO	2.39 (0.99)	2.40 (1.04)	2.38 (0.93)	0.6875

**Table 3:** Multivariable Analysis Results.

Effect		Estimate	*SE*	*p*-Value
PIU		0.15	0.01	<0.0001
Gender	Male	−0.19	0.04	<0.0001
PIU × Gender (male)		0.03	0.01	<0.0001
Age (years)	12	0.01	0.04	0.7538
	13	0.02	0.04	0.6069
	14	0.02	0.04	0.6566
	15	Reference		
	16	0.00	0.04	0.9874
	17	−0.05	0.04	0.2196
Race	White/Caucasian	0.12	0.03	0.0003
	Black/African American	0.11	0.04	0.0074
	Other	Reference		

Effect size of PIU × gender interaction is 3.

## Data Availability

The data presented in this study is available from the corresponding author upon reasonable request. The data is not publicly available due to restrictions imposed by the academic institution.

## Abbreviations

The following abbreviations are used in this manuscript:

PIU	Problematic Internet Use
FoMO	Fear of Missing Out
PRIUSS	Problematic and Risky Internet Use Screening Scale
